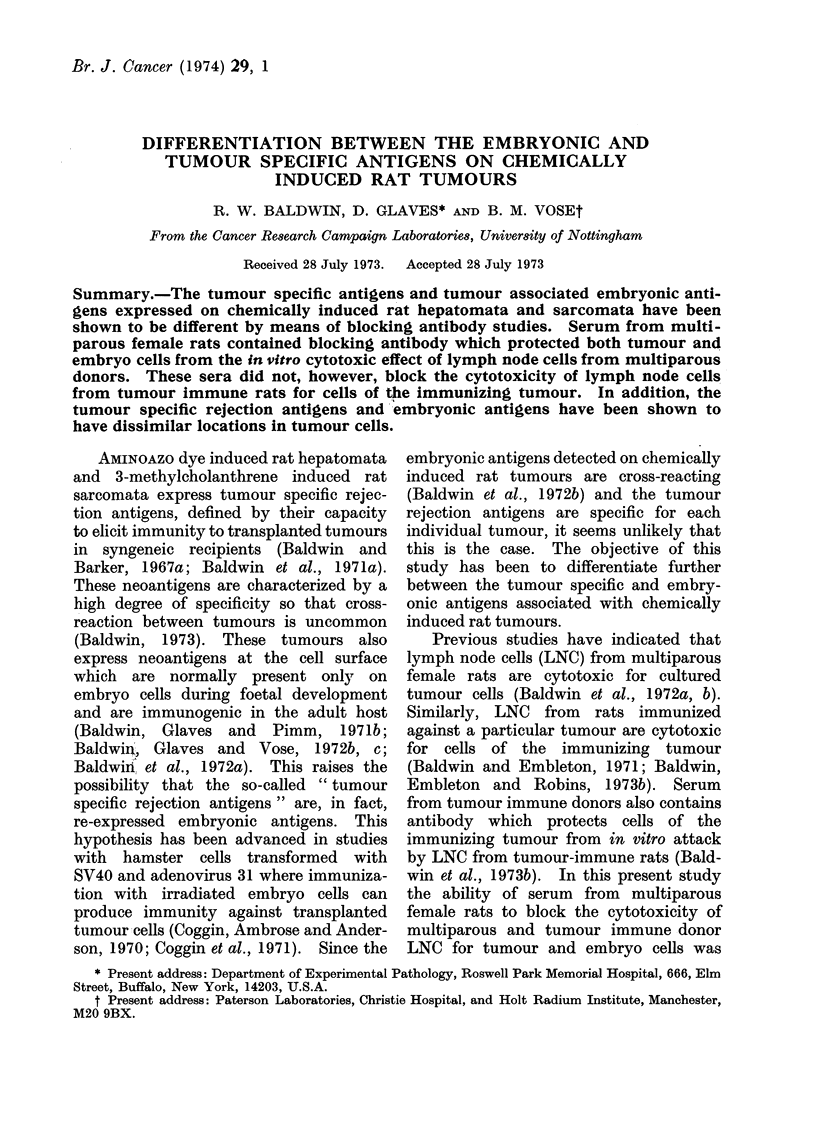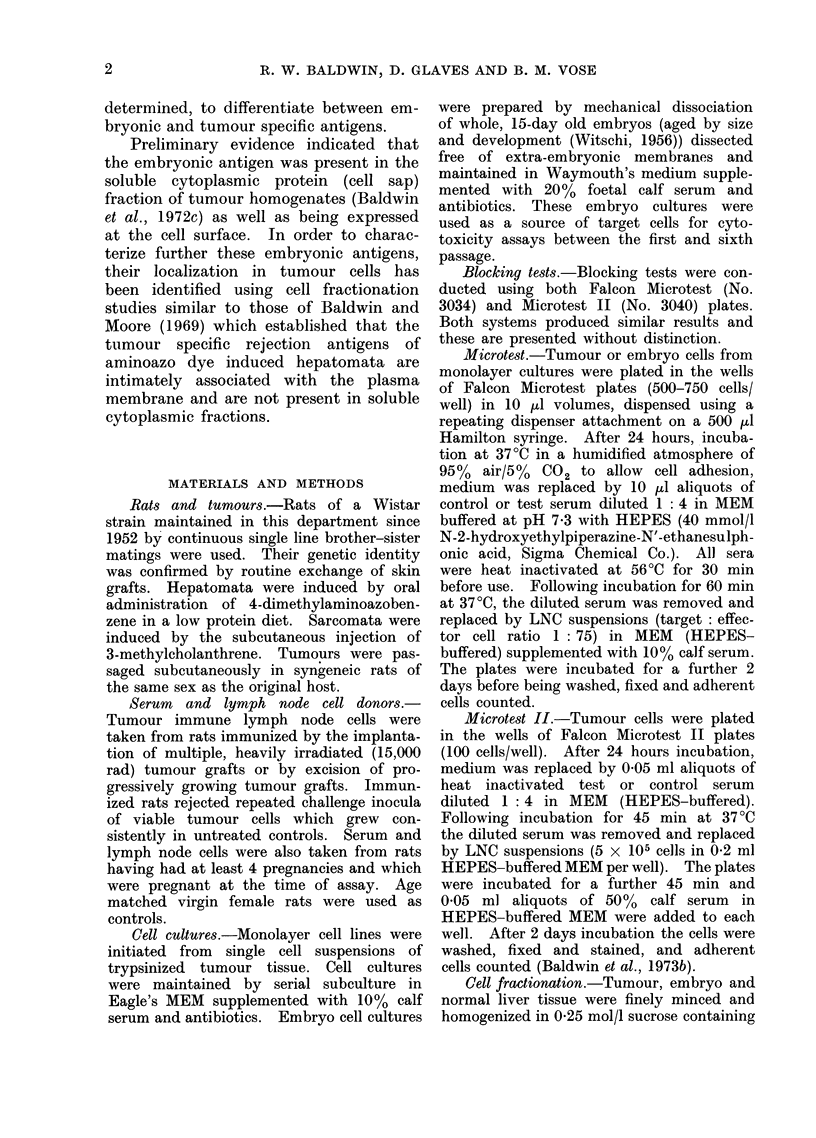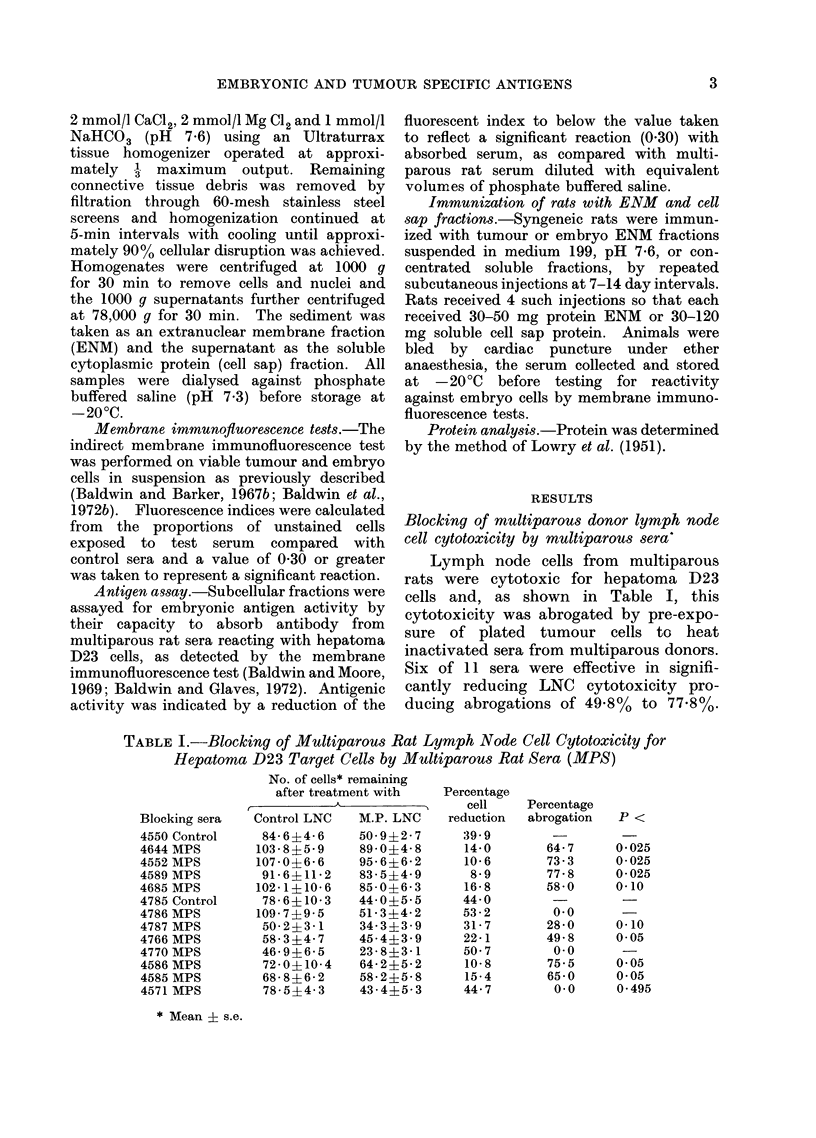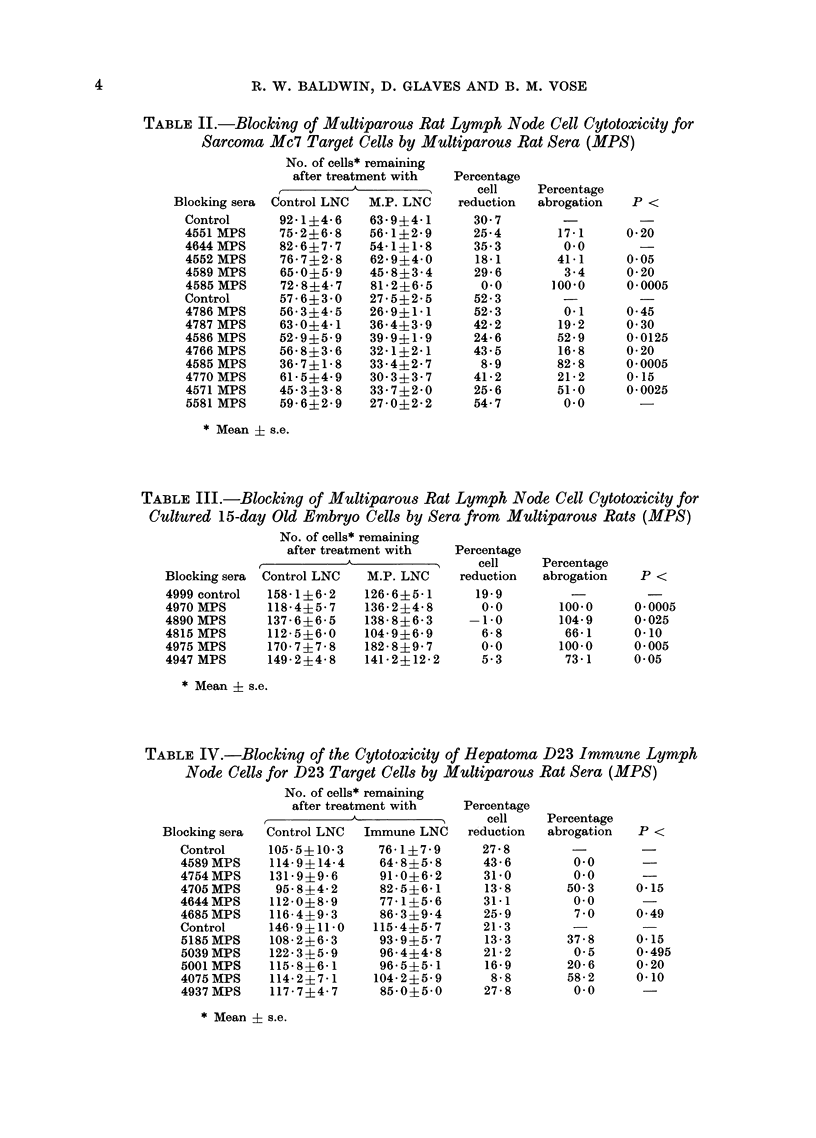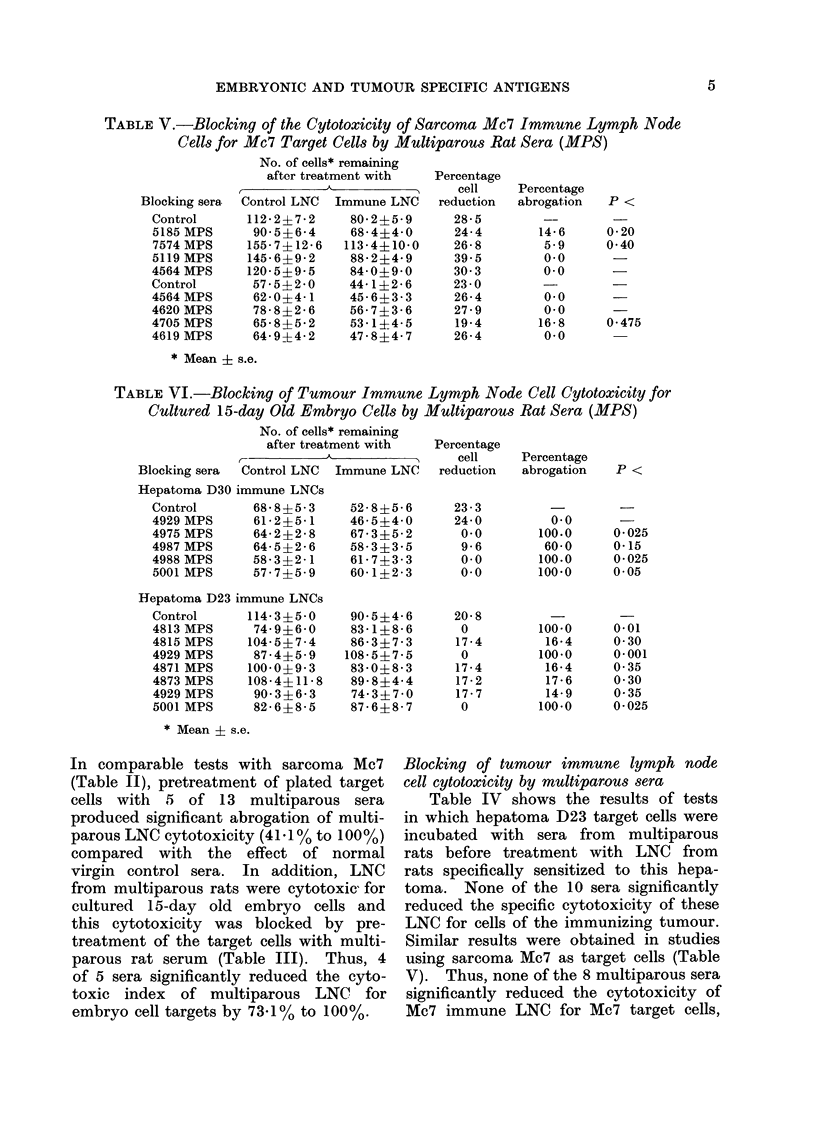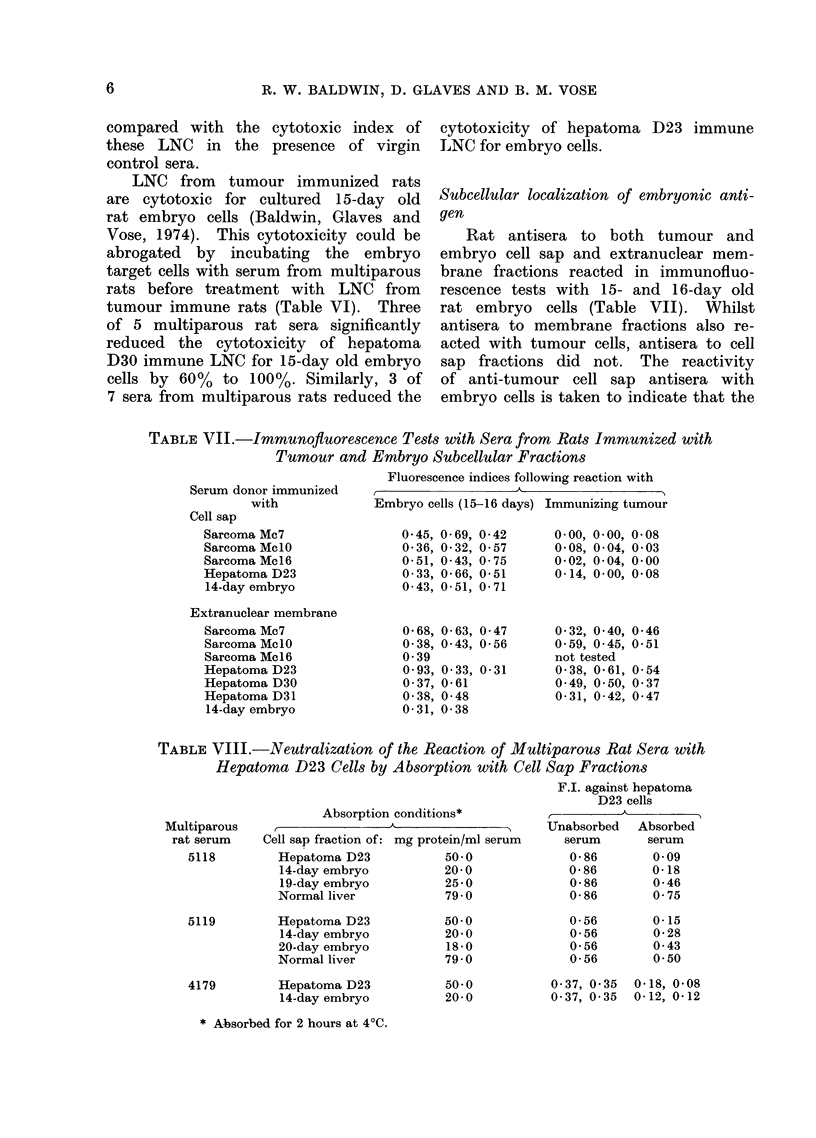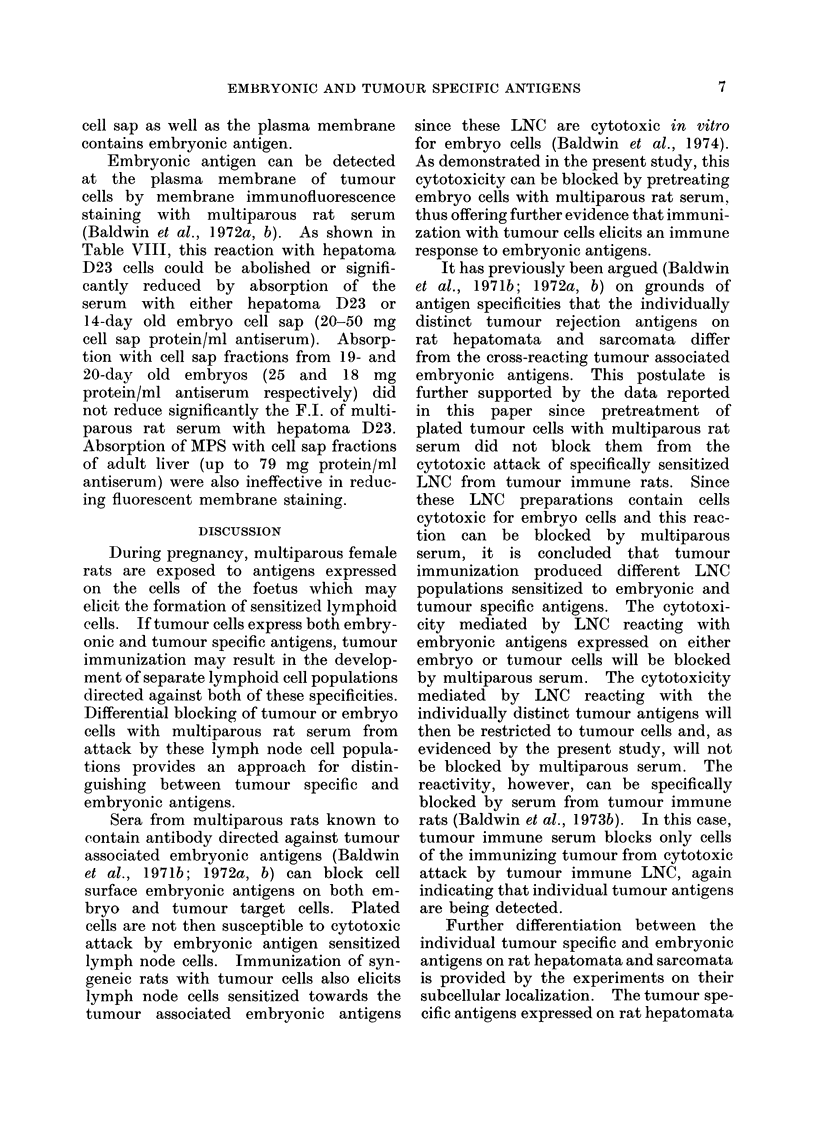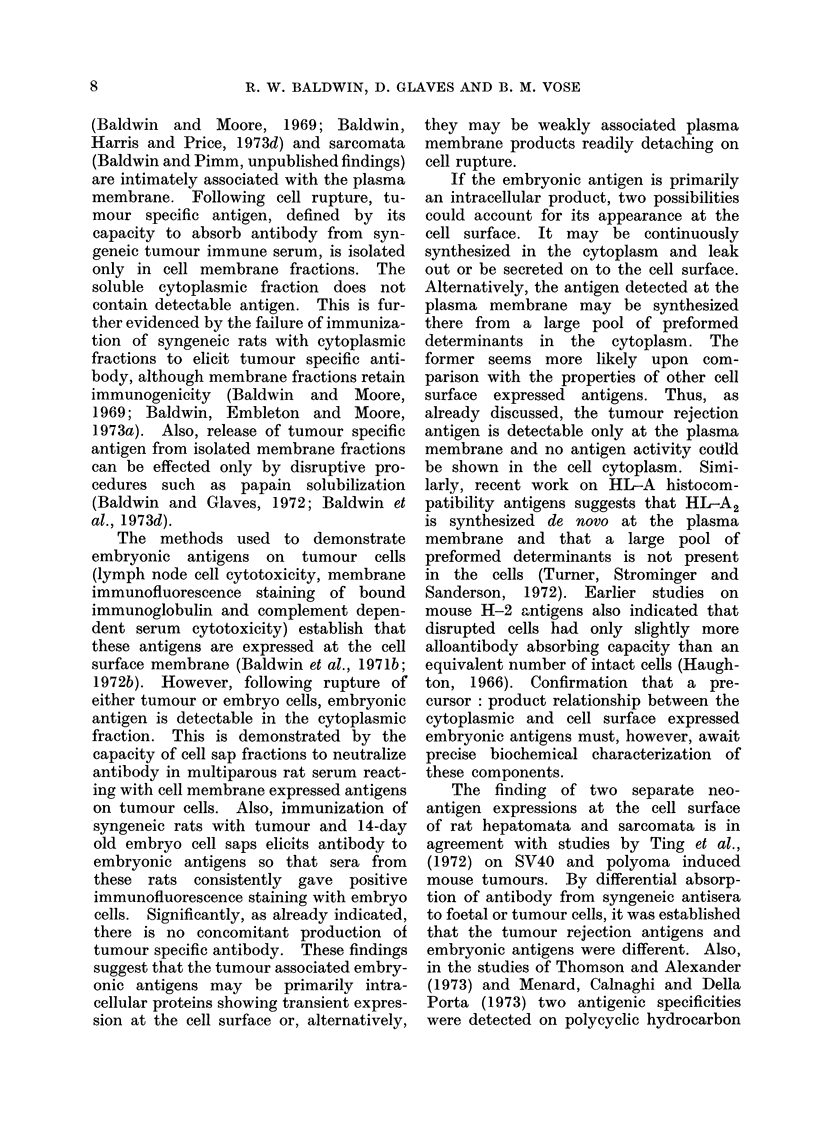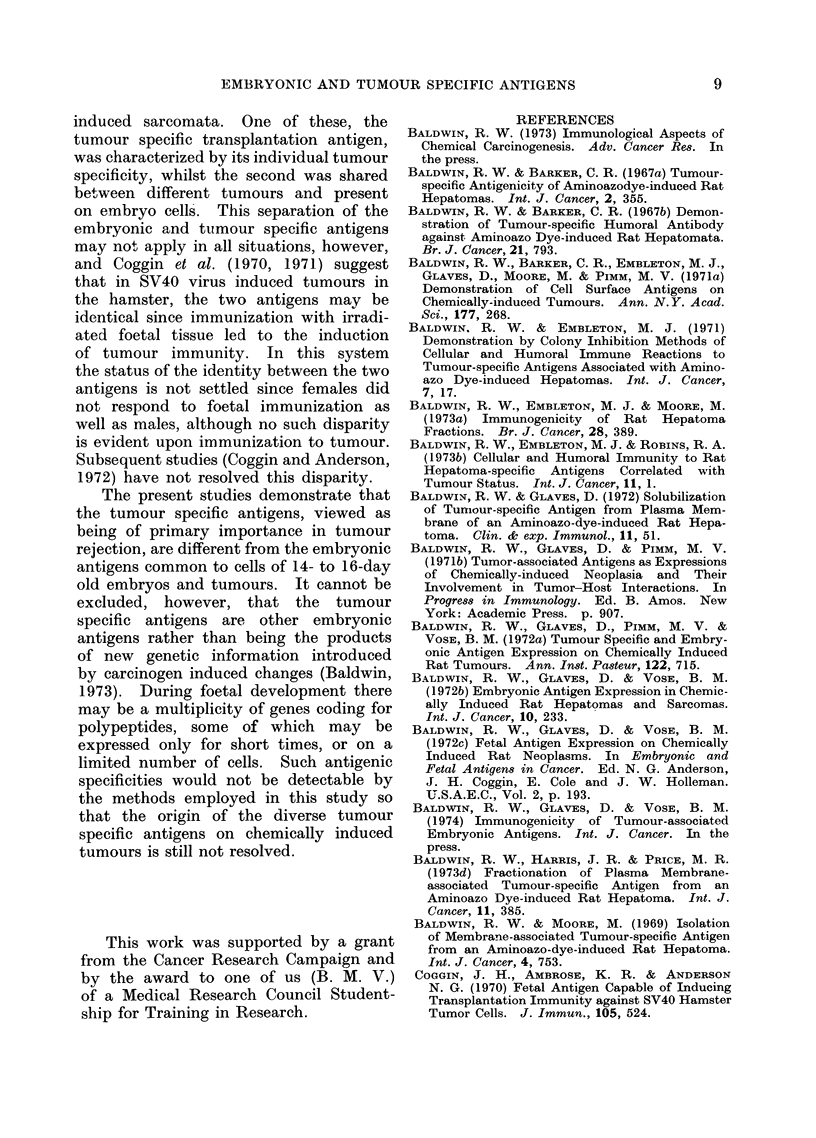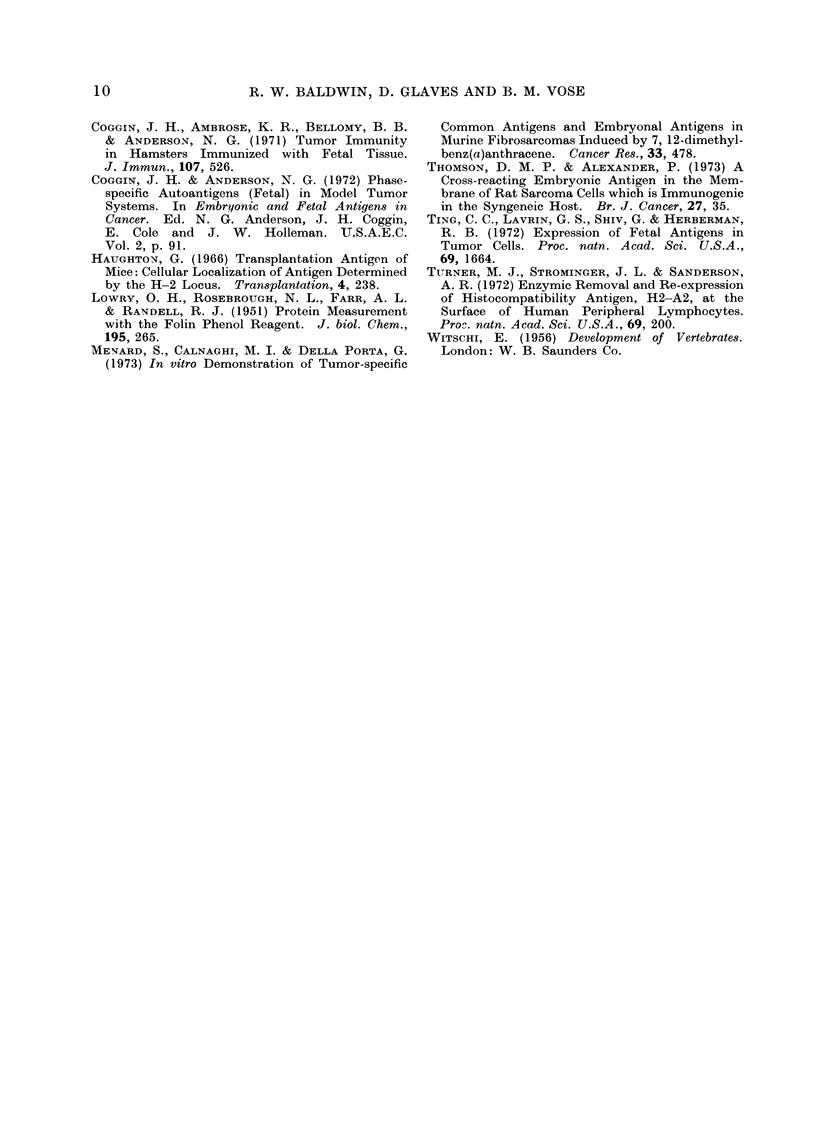# Differentiation Between the Embryonic and Tumour Specific Antigens on Chemically Induced Rat Tumours

**DOI:** 10.1038/bjc.1974.1

**Published:** 1974-01

**Authors:** R. W. Baldwin, D. Glaves, B. M. Vose

## Abstract

The tumour specific antigens and tumour associated embryonic antigens expressed on chemically induced rat hepatomata and sarcomata have been shown to be different by means of blocking antibody studies. Serum from multiparous female rats contained blocking antibody which protected both tumour and embryo cells from the *in vitro* cytotoxic effect of lymph node cells from multiparous donors. These sera did not, however, block the cytotoxicity of lymph node cells from tumour immune rats for cells of the immunizing tumour. In addition, the tumour specific rejection antigens and embryonic antigens have been shown to have dissimilar locations in tumour cells.


*Br. J. Cancer* (1974) **29**, 1

### DIFFERENTIATION BETWEEN THE EMBRYONIC AND TUMOUR SPECIFIC ANTIGENS ON CHEMICALLY INDUCED RAT TUMOURS

R. W. BALDWIN, D. GLAVES* AND B. M. VOSE†

*From the Cancer Research Campaign Laboratories, University of Nottingham*

Received 28 July 1973. Accepted 28 July 1973

**Summary.—The tumour specific antigens and tumour associated embryonic antigens expressed on chemically induced rat hepatomata and sarcomata have been shown to be different by means of blocking antibody studies. Serum from multiparous female rats contained blocking antibody which protected both tumour and embryo cells from the *in vitro* cytotoxic effect of lymph node cells from multiparous donors. These sera did not, however, block the cytotoxicity of lymph node cells from tumour immune rats for cells of the immunizing tumour. In addition, the tumour specific rejection antigens and embryonic antigens have been shown to have dissimilar locations in tumour cells.**

AMINOAZO dye induced rat hepatomata and 3-methylcholanthrene induced rat sarcomata express tumour specific rejection antigens, defined by their capacity to elicit immunity to transplanted tumours in syngeneic recipients (Baldwin and Barker, 1967*a*; Baldwin *et al.*, 1971*a*). These neoantigens are characterized by a high degree of specificity so that cross-reaction between tumours is uncommon (Baldwin, 1973). These tumours also express neoantigens at the cell surface which are normally present only on embryo cells during foetal development and are immunogenic in the adult host (Baldwin, Glaves and Pimm, 1971*b*; Baldwin, Glaves and Vose, 1972*b*, *c*; Baldwin *et al.*, 1972*a*). This raises the possibility that the so-called " tumour specific rejection antigens " are, in fact, re-expressed embryonic antigens. This hypothesis has been advanced in studies with hamster cells transformed with SV40 and adenovirus 31 where immunization with irradiated embryo cells can produce immunity against transplanted tumour cells (Coggin, Ambrose and Anderson, 1970; Coggin *et al.*, 1971). Since the embryonic antigens detected on chemically induced rat tumours are cross-reacting (Baldwin *et al.*, 1972*b*) and the tumour rejection antigens are specific for each individual tumour, it seems unlikely that this is the case. The objective of this study has been to differentiate further between the tumour specific and embryonic antigens associated with chemically induced rat tumours.

Previous studies have indicated that lymph node cells (LNC) from multiparous female rats are cytotoxic for cultured tumour cells (Baldwin *et al.*, 1972*a*, *b*). Similarly, LNC from rats immunized against a particular tumour are cytotoxic for cells of the immunizing tumour (Baldwin and Embleton, 1971; Baldwin, Embleton and Robins, 1973*b*). Serum from tumour immune donors also contains antibody which protects cells of the immunizing tumour from *in vitro* attack by LNC from tumour-immune rats (Baldwin *et al.*, 1973*b*). In this present study the ability of serum from multiparous female rats to block the cytotoxicity of multiparous and tumour immune donor LNC for tumour and embryo cells was

* Present address: Department of Experimental Pathology, Roswell Park Memorial Hospital, 666, Elm Street, Buffalo, New York, 14203, U.S.A.

† Present address: Paterson Laboratories, Christie Hospital, and Holt Radium Institute, Manchester, M20 9BX.

determined, to differentiate between embryonic and tumour specific antigens.

Preliminary evidence indicated that the embryonic antigen was present in the soluble cytoplasmic protein (cell sap) fraction of tumour homogenates (Baldwin *et al.*, 1972*c*) as well as being expressed at the cell surface. In order to characterize further these embryonic antigens, their localization in tumour cells has been identified using cell fractionation studies similar to those of Baldwin and Moore (1969) which established that the tumour specific rejection antigens of aminoazo dye induced hepatomata are intimately associated with the plasma membrane and are not present in soluble cytoplasmic fractions.

### MATERIALS AND METHODS

*Rats and tumours.*—Rats of a Wistar strain maintained in this department since 1952 by continuous single line brother–sister matings were used. Their genetic identity was confirmed by routine exchange of skin grafts. Hepatomata were induced by oral administration of 4-dimethylaminoazobenzene in a low protein diet. Sarcomata were induced by the subcutaneous injection of 3-methylcholanthrene. Tumours were passaged subcutaneously in syngeneic rats of the same sex as the original host.

*Serum and lymph node cell donors.*—Tumour immune lymph node cells were taken from rats immunized by the implantation of multiple, heavily irradiated (15,000 rad) tumour grafts or by excision of progressively growing tumour grafts. Immunized rats rejected repeated challenge inocula of viable tumour cells which grew consistently in untreated controls. Serum and lymph node cells were also taken from rats having had at least 4 pregnancies and which were pregnant at the time of assay. Age matched virgin female rats were used as controls.

*Cell cultures.*—Monolayer cell lines were initiated from single cell suspensions of trypsinized tumour tissue. Cell cultures were maintained by serial subculture in Eagle's MEM supplemented with 10% calf serum and antibiotics. Embryo cell cultures were prepared by mechanical dissociation of whole, 15-day old embryos (aged by size and development (Witschi, 1956)) dissected free of extra-embryonic membranes and maintained in Waymouth's medium supplemented with 20% foetal calf serum and antibiotics. These embryo cultures were used as a source of target cells for cytotoxicity assays between the first and sixth passage.

*Blocking tests.*—Blocking tests were conducted using both Falcon Microtest (No. 3034) and Microtest II (No. 3040) plates. Both systems produced similar results and these are presented without distinction.

*Microtest.*—Tumour or embryo cells from monolayer cultures were plated in the wells of Falcon Microtest plates (500–750 cells/well) in 10 $\mu$l volumes, dispensed using a repeating dispenser attachment on a 500 $\mu$l Hamilton syringe. After 24 hours, incubation at 37°C in a humidified atmosphere of 95% air/5% $CO_2$ to allow cell adhesion, medium was replaced by 10 $\mu$l aliquots of control or test serum diluted 1 : 4 in MEM buffered at pH 7·3 with HEPES (40 mmol/l N-2-hydroxyethylpiperazine-N′-ethanesulphonic acid, Sigma Chemical Co.). All sera were heat inactivated at 56°C for 30 min before use. Following incubation for 60 min at 37°C, the diluted serum was removed and replaced by LNC suspensions (target : effector cell ratio 1 : 75) in MEM (HEPES–buffered) supplemented with 10% calf serum. The plates were incubated for a further 2 days before being washed, fixed and adherent cells counted.

*Microtest II.*—Tumour cells were plated in the wells of Falcon Microtest II plates (100 cells/well). After 24 hours incubation, medium was replaced by 0·05 ml aliquots of heat inactivated test or control serum diluted 1 : 4 in MEM (HEPES–buffered). Following incubation for 45 min at 37°C the diluted serum was removed and replaced by LNC suspensions ($5 \times 10^5$ cells in 0·2 ml HEPES–buffered MEM per well). The plates were incubated for a further 45 min and 0·05 ml aliquots of 50% calf serum in HEPES–buffered MEM were added to each well. After 2 days incubation the cells were washed, fixed and stained, and adherent cells counted (Baldwin *et al.*, 1973*b*).

*Cell fractionation.*—Tumour, embryo and normal liver tissue were finely minced and homogenized in 0·25 mol/l sucrose containing

2 mmol/l $CaCl_2$, 2 mmol/l Mg $Cl_2$ and 1 mmol/l $NaHCO_3$ (pH 7·6) using an Ultraturrax tissue homogenizer operated at approximately $\frac{1}{3}$ maximum output. Remaining connective tissue debris was removed by filtration through 60-mesh stainless steel screens and homogenization continued at 5-min intervals with cooling until approximately 90% cellular disruption was achieved. Homogenates were centrifuged at 1000 *g* for 30 min to remove cells and nuclei and the 1000 *g* supernatants further centrifuged at 78,000 *g* for 30 min. The sediment was taken as an extranuclear membrane fraction (ENM) and the supernatant as the soluble cytoplasmic protein (cell sap) fraction. All samples were dialysed against phosphate buffered saline (pH 7·3) before storage at −20°C.

*Membrane immunofluorescence tests.*—The indirect membrane immunofluorescence test was performed on viable tumour and embryo cells in suspension as previously described (Baldwin and Barker, 1967*b*; Baldwin *et al.*, 1972*b*). Fluorescence indices were calculated from the proportions of unstained cells exposed to test serum compared with control sera and a value of 0·30 or greater was taken to represent a significant reaction.

*Antigen assay.*—Subcellular fractions were assayed for embryonic antigen activity by their capacity to absorb antibody from multiparous rat sera reacting with hepatoma D23 cells, as detected by the membrane immunofluorescence test (Baldwin and Moore, 1969; Baldwin and Glaves, 1972). Antigenic activity was indicated by a reduction of the fluorescent index to below the value taken to reflect a significant reaction (0·30) with absorbed serum, as compared with multiparous rat serum diluted with equivalent volumes of phosphate buffered saline.

*Immunization of rats with ENM and cell sap fractions.*—Syngeneic rats were immunized with tumour or embryo ENM fractions suspended in medium 199, pH 7·6, or concentrated soluble fractions, by repeated subcutaneous injections at 7–14 day intervals. Rats received 4 such injections so that each received 30–50 mg protein ENM or 30–120 mg soluble cell sap protein. Animals were bled by cardiac puncture under ether anaesthesia, the serum collected and stored at −20°C before testing for reactivity against embryo cells by membrane immunofluorescence tests.

*Protein analysis.*—Protein was determined by the method of Lowry *et al.* (1951).

### RESULTS

### *Blocking of multiparous donor lymph node cell cytotoxicity by multiparous sera*

Lymph node cells from multiparous rats were cytotoxic for hepatoma D23 cells and, as shown in Table I, this cytotoxicity was abrogated by pre-exposure of plated tumour cells to heat inactivated sera from multiparous donors. Six of 11 sera were effective in significantly reducing LNC cytotoxicity producing abrogations of 49·8% to 77·8%.

TABLE I.—*Blocking of Multiparous Rat Lymph Node Cell Cytotoxicity for Hepatoma D23 Target Cells by Multiparous Rat Sera (MPS)*

| Blocking sera | No. of cells* remaining after treatment with: Control LNC | M.P. LNC | Percentage cell reduction | Percentage abrogation | $P <$ |
|---|---|---|---|---|---|
| 4550 Control | 84·6±4·6 | 50·9±2·7 | 39·9 | — | — |
| 4644 MPS | 103·8±5·9 | 89·0±4·8 | 14·0 | 64·7 | 0·025 |
| 4552 MPS | 107·0±6·6 | 95·6±6·2 | 10·6 | 73·3 | 0·025 |
| 4589 MPS | 91·6±11·2 | 83·5±4·9 | 8·9 | 77·8 | 0·025 |
| 4685 MPS | 102·1±10·6 | 85·0±6·3 | 16·8 | 58·0 | 0·10 |
| 4785 Control | 78·6±10·3 | 44·0±5·5 | 44·0 | — | — |
| 4786 MPS | 109·7±9·5 | 51·3±4·2 | 53·2 | 0·0 | — |
| 4787 MPS | 50·2±3·1 | 34·3±3·9 | 31·7 | 28·0 | 0·10 |
| 4766 MPS | 58·3±4·7 | 45·4±3·9 | 22·1 | 49·8 | 0·05 |
| 4770 MPS | 46·9±6·5 | 23·8±3·1 | 50·7 | 0·0 | — |
| 4586 MPS | 72·0±10·4 | 64·2±5·2 | 10·8 | 75·5 | 0·05 |
| 4585 MPS | 68·8±6·2 | 58·2±5·8 | 15·4 | 65·0 | 0·05 |
| 4571 MPS | 78·5±4·3 | 43·4±5·3 | 44·7 | 0·0 | 0·495 |

* Mean ± s.e.

TABLE II.—*Blocking of Multiparous Rat Lymph Node Cell Cytotoxicity for Sarcoma Mc7 Target Cells by Multiparous Rat Sera (MPS)*

| Blocking sera | No. of cells* remaining after treatment with Control LNC | M.P. LNC | Percentage cell reduction | Percentage abrogation | $P <$ |
|---|---|---|---|---|---|
| Control | 92·1±4·6 | 63·9±4·1 | 30·7 | — | — |
| 4551 MPS | 75·2±6·8 | 56·1±2·9 | 25·4 | 17·1 | 0·20 |
| 4644 MPS | 82·6±7·7 | 54·1±1·8 | 35·3 | 0·0 | — |
| 4552 MPS | 76·7±2·8 | 62·9±4·0 | 18·1 | 41·1 | 0·05 |
| 4589 MPS | 65·0±5·9 | 45·8±3·4 | 29·6 | 3·4 | 0·20 |
| 4585 MPS | 72·8±4·7 | 81·2±6·5 | 0·0 | 100·0 | 0·0005 |
| Control | 57·6±3·0 | 27·5±2·5 | 52·3 | — | — |
| 4786 MPS | 56·3±4·5 | 26·9±1·1 | 52·3 | 0·1 | 0·45 |
| 4787 MPS | 63·0±4·1 | 36·4±3·9 | 42·2 | 19·2 | 0·30 |
| 4586 MPS | 52·9±5·9 | 39·9±1·9 | 24·6 | 52·9 | 0·0125 |
| 4766 MPS | 56·8±3·6 | 32·1±2·1 | 43·5 | 16·8 | 0·20 |
| 4585 MPS | 36·7±1·8 | 33·4±2·7 | 8·9 | 82·8 | 0·0005 |
| 4770 MPS | 61·5±4·9 | 30·3±3·7 | 41·2 | 21·2 | 0·15 |
| 4571 MPS | 45·3±3·8 | 33·7±2·0 | 25·6 | 51·0 | 0·0025 |
| 5581 MPS | 59·6±2·9 | 27·0±2·2 | 54·7 | 0·0 | — |

* Mean ± s.e.

TABLE III.—*Blocking of Multiparous Rat Lymph Node Cell Cytotoxicity for Cultured 15-day Old Embryo Cells by Sera from Multiparous Rats (MPS)*

| Blocking sera | No. of cells* remaining after treatment with Control LNC | M.P. LNC | Percentage cell reduction | Percentage abrogation | $P <$ |
|---|---|---|---|---|---|
| 4999 control | 158·1±6·2 | 126·6±5·1 | 19·9 | — | — |
| 4970 MPS | 118·4±5·7 | 136·2±4·8 | 0·0 | 100·0 | 0·0005 |
| 4890 MPS | 137·6±6·5 | 138·8±6·3 | −1·0 | 104·9 | 0·025 |
| 4815 MPS | 112·5±6·0 | 104·9±6·9 | 6·8 | 66·1 | 0·10 |
| 4975 MPS | 170·7±7·8 | 182·8±9·7 | 0·0 | 100·0 | 0·005 |
| 4947 MPS | 149·2±4·8 | 141·2±12·2 | 5·3 | 73·1 | 0·05 |

* Mean ± s.e.

TABLE IV.—*Blocking of the Cytotoxicity of Hepatoma D23 Immune Lymph Node Cells for D23 Target Cells by Multiparous Rat Sera (MPS)*

| Blocking sera | No. of cells* remaining after treatment with Control LNC | Immune LNC | Percentage cell reduction | Percentage abrogation | $P <$ |
|---|---|---|---|---|---|
| Control | 105·5±10·3 | 76·1±7·9 | 27·8 | — | — |
| 4589 MPS | 114·9±14·4 | 64·8±5·8 | 43·6 | 0·0 | — |
| 4754 MPS | 131·9±9·6 | 91·0±6·2 | 31·0 | 0·0 | — |
| 4705 MPS | 95·8±4·2 | 82·5±6·1 | 13·8 | 50·3 | 0·15 |
| 4644 MPS | 112·0±8·9 | 77·1±5·6 | 31·1 | 0·0 | — |
| 4685 MPS | 116·4±9·3 | 86·3±9·4 | 25·9 | 7·0 | 0·49 |
| Control | 146·9±11·0 | 115·4±5·7 | 21·3 | — | — |
| 5185 MPS | 108·2±6·3 | 93·9±5·7 | 13·3 | 37·8 | 0·15 |
| 5039 MPS | 122·3±5·9 | 96·4±4·8 | 21·2 | 0·5 | 0·495 |
| 5001 MPS | 115·8±6·1 | 96·5±5·1 | 16·9 | 20·6 | 0·20 |
| 4075 MPS | 114·2±7·1 | 104·2±5·9 | 8·8 | 58·2 | 0·10 |
| 4937 MPS | 117·7±4·7 | 85·0±5·0 | 27·8 | 0·0 | — |

* Mean ± s.e.

TABLE V.—*Blocking of the Cytotoxicity of Sarcoma Mc7 Immune Lymph Node Cells for Mc7 Target Cells by Multiparous Rat Sera (MPS)*

| Blocking sera | No. of cells* remaining after treatment with: Control LNC | Immune LNC | Percentage cell reduction | Percentage abrogation | $P <$ |
|---|---|---|---|---|---|
| Control | 112·2±7·2 | 80·2±5·9 | 28·5 | — | — |
| 5185 MPS | 90·5±6·4 | 68·4±4·0 | 24·4 | 14·6 | 0·20 |
| 7574 MPS | 155·7±12·6 | 113·4±10·0 | 26·8 | 5·9 | 0·40 |
| 5119 MPS | 145·6±9·2 | 88·2±4·9 | 39·5 | 0·0 | — |
| 4564 MPS | 120·5±9·5 | 84·0±9·0 | 30·3 | 0·0 | — |
| Control | 57·5±2·0 | 44·1±2·6 | 23·0 | — | — |
| 4564 MPS | 62·0±4·1 | 45·6±3·3 | 26·4 | 0·0 | — |
| 4620 MPS | 78·8±2·6 | 56·7±3·6 | 27·9 | 0·0 | — |
| 4705 MPS | 65·8±5·2 | 53·1±4·5 | 19·4 | 16·8 | 0·475 |
| 4619 MPS | 64·9±4·2 | 47·8±4·7 | 26·4 | 0·0 | — |

* Mean ± s.e.

TABLE VI.—*Blocking of Tumour Immune Lymph Node Cell Cytotoxicity for Cultured 15-day Old Embryo Cells by Multiparous Rat Sera (MPS)*

| Blocking sera | No. of cells* remaining after treatment with: Control LNC | Immune LNC | Percentage cell reduction | Percentage abrogation | $P <$ |
|---|---|---|---|---|---|
| Hepatoma D30 immune LNCs | | | | | |
| Control | 68·8±5·3 | 52·8±5·6 | 23·3 | — | — |
| 4929 MPS | 61·2±5·1 | 46·5±4·0 | 24·0 | 0·0 | — |
| 4975 MPS | 64·2±2·8 | 67·3±5·2 | 0·0 | 100·0 | 0·025 |
| 4987 MPS | 64·5±2·6 | 58·3±3·5 | 9·6 | 60·0 | 0·15 |
| 4988 MPS | 58·3±2·1 | 61·7±3·3 | 0·0 | 100·0 | 0·025 |
| 5001 MPS | 57·7±5·9 | 60·1±2·3 | 0·0 | 100·0 | 0·05 |
| Hepatoma D23 immune LNCs | | | | | |
| Control | 114·3±5·0 | 90·5±4·6 | 20·8 | — | — |
| 4813 MPS | 74·9±6·0 | 83·1±8·6 | 0 | 100·0 | 0·01 |
| 4815 MPS | 104·5±7·4 | 86·3±7·3 | 17·4 | 16·4 | 0·30 |
| 4929 MPS | 87·4±5·9 | 108·5±7·5 | 0 | 100·0 | 0·001 |
| 4871 MPS | 100·0±9·3 | 83·0±8·3 | 17·4 | 16·4 | 0·35 |
| 4873 MPS | 108·4±11·8 | 89·8±4·4 | 17·2 | 17·6 | 0·30 |
| 4929 MPS | 90·3±6·3 | 74·3±7·0 | 17·7 | 14·9 | 0·35 |
| 5001 MPS | 82·6±8·5 | 87·6±8·7 | 0 | 100·0 | 0·025 |

* Mean ± s.e.

In comparable tests with sarcoma Mc7 (Table II), pretreatment of plated target cells with 5 of 13 multiparous sera produced significant abrogation of multiparous LNC cytotoxicity (41·1% to 100%) compared with the effect of normal virgin control sera. In addition, LNC from multiparous rats were cytotoxic for cultured 15-day old embryo cells and this cytotoxicity was blocked by pretreatment of the target cells with multiparous rat serum (Table III). Thus, 4 of 5 sera significantly reduced the cytotoxic index of multiparous LNC for embryo cell targets by 73·1% to 100%.

*Blocking of tumour immune lymph node cell cytotoxicity by multiparous sera*

Table IV shows the results of tests in which hepatoma D23 target cells were incubated with sera from multiparous rats before treatment with LNC from rats specifically sensitized to this hepatoma. None of the 10 sera significantly reduced the specific cytotoxicity of these LNC for cells of the immunizing tumour. Similar results were obtained in studies using sarcoma Mc7 as target cells (Table V). Thus, none of the 8 multiparous sera significantly reduced the cytotoxicity of Mc7 immune LNC for Mc7 target cells, compared with the cytotoxic index of these LNC in the presence of virgin control sera.

LNC from tumour immunized rats are cytotoxic for cultured 15-day old rat embryo cells (Baldwin, Glaves and Vose, 1974). This cytotoxicity could be abrogated by incubating the embryo target cells with serum from multiparous rats before treatment with LNC from tumour immune rats (Table VI). Three of 5 multiparous rat sera significantly reduced the cytotoxicity of hepatoma D30 immune LNC for 15-day old embryo cells by 60% to 100%. Similarly, 3 of 7 sera from multiparous rats reduced the cytotoxicity of hepatoma D23 immune LNC for embryo cells.

*Subcellular localization of embryonic antigen*

Rat antisera to both tumour and embryo cell sap and extranuclear membrane fractions reacted in immunofluorescence tests with 15- and 16-day old rat embryo cells (Table VII). Whilst antisera to membrane fractions also reacted with tumour cells, antisera to cell sap fractions did not. The reactivity of anti-tumour cell sap antisera with embryo cells is taken to indicate that the

TABLE VII.—*Immunofluorescence Tests with Sera from Rats Immunized with Tumour and Embryo Subcellular Fractions*

| Serum donor immunized with | Fluorescence indices following reaction with: Embryo cells (15–16 days) | Immunizing tumour |
|---|---|---|
| Cell sap | | |
| Sarcoma Mc7 | 0·45, 0·69, 0·42 | 0·00, 0·00, 0·08 |
| Sarcoma Mc10 | 0·36, 0·32, 0·57 | 0·08, 0·04, 0·03 |
| Sarcoma Mc16 | 0·51, 0·43, 0·75 | 0·02, 0·04, 0·00 |
| Hepatoma D23 | 0·33, 0·66, 0·51 | 0·14, 0·00, 0·08 |
| 14-day embryo | 0·43, 0·51, 0·71 | |
| Extranuclear membrane | | |
| Sarcoma Mc7 | 0·68, 0·63, 0·47 | 0·32, 0·40, 0·46 |
| Sarcoma Mc10 | 0·38, 0·43, 0·56 | 0·59, 0·45, 0·51 |
| Sarcoma Mc16 | 0·39 | not tested |
| Hepatoma D23 | 0·93, 0·33, 0·31 | 0·38, 0·61, 0·54 |
| Hepatoma D30 | 0·37, 0·61 | 0·49, 0·50, 0·37 |
| Hepatoma D31 | 0·38, 0·48 | 0·31, 0·42, 0·47 |
| 14-day embryo | 0·31, 0·38 | |

TABLE VIII.—*Neutralization of the Reaction of Multiparous Rat Sera with Hepatoma D23 Cells by Absorption with Cell Sap Fractions*

| Multiparous rat serum | Absorption conditions*: Cell sap fraction of: | mg protein/ml serum | F.I. against hepatoma D23 cells: Unabsorbed serum | Absorbed serum |
|---|---|---|---|---|
| 5118 | Hepatoma D23 | 50·0 | 0·86 | 0·09 |
| | 14-day embryo | 20·0 | 0·86 | 0·18 |
| | 19-day embryo | 25·0 | 0·86 | 0·46 |
| | Normal liver | 79·0 | 0·86 | 0·75 |
| 5119 | Hepatoma D23 | 50·0 | 0·56 | 0·15 |
| | 14-day embryo | 20·0 | 0·56 | 0·28 |
| | 20-day embryo | 18·0 | 0·56 | 0·43 |
| | Normal liver | 79·0 | 0·56 | 0·50 |
| 4179 | Hepatoma D23 | 50·0 | 0·37, 0·35 | 0·18, 0·08 |
| | 14-day embryo | 20·0 | 0·37, 0·35 | 0·12, 0·12 |

* Absorbed for 2 hours at 4°C.

cell sap as well as the plasma membrane contains embryonic antigen.

Embryonic antigen can be detected at the plasma membrane of tumour cells by membrane immunofluorescence staining with multiparous rat serum (Baldwin *et al.*, 1972*a*, *b*). As shown in Table VIII, this reaction with hepatoma D23 cells could be abolished or significantly reduced by absorption of the serum with either hepatoma D23 or 14-day old embryo cell sap (20–50 mg cell sap protein/ml antiserum). Absorption with cell sap fractions from 19- and 20-day old embryos (25 and 18 mg protein/ml antiserum respectively) did not reduce significantly the F.I. of multiparous rat serum with hepatoma D23. Absorption of MPS with cell sap fractions of adult liver (up to 79 mg protein/ml antiserum) were also ineffective in reducing fluorescent membrane staining.

### DISCUSSION

During pregnancy, multiparous female rats are exposed to antigens expressed on the cells of the foetus which may elicit the formation of sensitized lymphoid cells. If tumour cells express both embryonic and tumour specific antigens, tumour immunization may result in the development of separate lymphoid cell populations directed against both of these specificities. Differential blocking of tumour or embryo cells with multiparous rat serum from attack by these lymph node cell populations provides an approach for distinguishing between tumour specific and embryonic antigens.

Sera from multiparous rats known to contain antibody directed against tumour associated embryonic antigens (Baldwin *et al.*, 1971*b*; 1972*a*, *b*) can block cell surface embryonic antigens on both embryo and tumour target cells. Plated cells are not then susceptible to cytotoxic attack by embryonic antigen sensitized lymph node cells. Immunization of syngeneic rats with tumour cells also elicits lymph node cells sensitized towards the tumour associated embryonic antigens since these LNC are cytotoxic *in vitro* for embryo cells (Baldwin *et al.*, 1974). As demonstrated in the present study, this cytotoxicity can be blocked by pretreating embryo cells with multiparous rat serum, thus offering further evidence that immunization with tumour cells elicits an immune response to embryonic antigens.

It has previously been argued (Baldwin *et al.*, 1971*b*; 1972*a*, *b*) on grounds of antigen specificities that the individually distinct tumour rejection antigens on rat hepatomata and sarcomata differ from the cross-reacting tumour associated embryonic antigens. This postulate is further supported by the data reported in this paper since pretreatment of plated tumour cells with multiparous rat serum did not block them from the cytotoxic attack of specifically sensitized LNC from tumour immune rats. Since these LNC preparations contain cells cytotoxic for embryo cells and this reaction can be blocked by multiparous serum, it is concluded that tumour immunization produced different LNC populations sensitized to embryonic and tumour specific antigens. The cytotoxicity mediated by LNC reacting with embryonic antigens expressed on either embryo or tumour cells will be blocked by multiparous serum. The cytotoxicity mediated by LNC reacting with the individually distinct tumour antigens will then be restricted to tumour cells and, as evidenced by the present study, will not be blocked by multiparous serum. The reactivity, however, can be specifically blocked by serum from tumour immune rats (Baldwin *et al.*, 1973*b*). In this case, tumour immune serum blocks only cells of the immunizing tumour from cytotoxic attack by tumour immune LNC, again indicating that individual tumour antigens are being detected.

Further differentiation between the individual tumour specific and embryonic antigens on rat hepatomata and sarcomata is provided by the experiments on their subcellular localization. The tumour specific antigens expressed on rat hepatomata

(Baldwin and Moore, 1969; Baldwin, Harris and Price, 1973*d*) and sarcomata (Baldwin and Pimm, unpublished findings) are intimately associated with the plasma membrane. Following cell rupture, tumour specific antigen, defined by its capacity to absorb antibody from syngeneic tumour immune serum, is isolated only in cell membrane fractions. The soluble cytoplasmic fraction does not contain detectable antigen. This is further evidenced by the failure of immunization of syngeneic rats with cytoplasmic fractions to elicit tumour specific antibody, although membrane fractions retain immunogenicity (Baldwin and Moore, 1969; Baldwin, Embleton and Moore, 1973*a*). Also, release of tumour specific antigen from isolated membrane fractions can be effected only by disruptive procedures such as papain solubilization (Baldwin and Glaves, 1972; Baldwin *et al.*, 1973*d*).

The methods used to demonstrate embryonic antigens on tumour cells (lymph node cell cytotoxicity, membrane immunofluorescence staining of bound immunoglobulin and complement dependent serum cytotoxicity) establish that these antigens are expressed at the cell surface membrane (Baldwin *et al.*, 1971*b*; 1972*b*). However, following rupture of either tumour or embryo cells, embryonic antigen is detectable in the cytoplasmic fraction. This is demonstrated by the capacity of cell sap fractions to neutralize antibody in multiparous rat serum reacting with cell membrane expressed antigens on tumour cells. Also, immunization of syngeneic rats with tumour and 14-day old embryo cell saps elicits antibody to embryonic antigens so that sera from these rats consistently gave positive immunofluorescence staining with embryo cells. Significantly, as already indicated, there is no concomitant production of tumour specific antibody. These findings suggest that the tumour associated embryonic antigens may be primarily intracellular proteins showing transient expression at the cell surface or, alternatively, they may be weakly associated plasma membrane products readily detaching on cell rupture.

If the embryonic antigen is primarily an intracellular product, two possibilities could account for its appearance at the cell surface. It may be continuously synthesized in the cytoplasm and leak out or be secreted on to the cell surface. Alternatively, the antigen detected at the plasma membrane may be synthesized there from a large pool of preformed determinants in the cytoplasm. The former seems more likely upon comparison with the properties of other cell surface expressed antigens. Thus, as already discussed, the tumour rejection antigen is detectable only at the plasma membrane and no antigen activity could be shown in the cell cytoplasm. Similarly, recent work on HL–A histocompatibility antigens suggests that HL–$A_2$ is synthesized *de novo* at the plasma membrane and that a large pool of preformed determinants is not present in the cells (Turner, Strominger and Sanderson, 1972). Earlier studies on mouse H–2 antigens also indicated that disrupted cells had only slightly more alloantibody absorbing capacity than an equivalent number of intact cells (Haughton, 1966). Confirmation that a precursor : product relationship between the cytoplasmic and cell surface expressed embryonic antigens must, however, await precise biochemical characterization of these components.

The finding of two separate neoantigen expressions at the cell surface of rat hepatomata and sarcomata is in agreement with studies by Ting *et al.*, (1972) on SV40 and polyoma induced mouse tumours. By differential absorption of antibody from syngeneic antisera to foetal or tumour cells, it was established that the tumour rejection antigens and embryonic antigens were different. Also, in the studies of Thomson and Alexander (1973) and Menard, Calnaghi and Della Porta (1973) two antigenic specificities were detected on polycyclic hydrocarbon induced sarcomata. One of these, the tumour specific transplantation antigen, was characterized by its individual tumour specificity, whilst the second was shared between different tumours and present on embryo cells. This separation of the embryonic and tumour specific antigens may not apply in all situations, however, and Coggin *et al.* (1970, 1971) suggest that in SV40 virus induced tumours in the hamster, the two antigens may be identical since immunization with irradiated foetal tissue led to the induction of tumour immunity. In this system the status of the identity between the two antigens is not settled since females did not respond to foetal immunization as well as males, although no such disparity is evident upon immunization to tumour. Subsequent studies (Coggin and Anderson, 1972) have not resolved this disparity.

The present studies demonstrate that the tumour specific antigens, viewed as being of primary importance in tumour rejection, are different from the embryonic antigens common to cells of 14- to 16-day old embryos and tumours. It cannot be excluded, however, that the tumour specific antigens are other embryonic antigens rather than being the products of new genetic information introduced by carcinogen induced changes (Baldwin, 1973). During foetal development there may be a multiplicity of genes coding for polypeptides, some of which may be expressed only for short times, or on a limited number of cells. Such antigenic specificities would not be detectable by the methods employed in this study so that the origin of the diverse tumour specific antigens on chemically induced tumours is still not resolved.

This work was supported by a grant from the Cancer Research Campaign and by the award to one of us (B. M. V.) of a Medical Research Council Studentship for Training in Research.

REFERENCES

BALDWIN, R. W. (1973) Immunological Aspects of Chemical Carcinogenesis. *Adv. Cancer Res.* In the press.

BALDWIN, R. W. & BARKER, C. R. (1967*a*) Tumour-specific Antigenicity of Aminoazodye-induced Rat Hepatomas. *Int. J. Cancer*, **2**, 355.

BALDWIN, R. W. & BARKER, C. R. (1967*b*) Demonstration of Tumour-specific Humoral Antibody against Aminoazo Dye-induced Rat Hepatomata. *Br. J. Cancer*, **21**, 793.

BALDWIN, R. W., BARKER, C. R., EMBLETON, M. J., GLAVES, D., MOORE, M. & PIMM, M. V. (1971*a*) Demonstration of Cell Surface Antigens on Chemically-induced Tumours. *Ann. N.Y. Acad. Sci.*, **177**, 268.

BALDWIN, R. W. & EMBLETON, M. J. (1971) Demonstration by Colony Inhibition Methods of Cellular and Humoral Immune Reactions to Tumour-specific Antigens Associated with Aminoazo Dye-induced Hepatomas. *Int. J. Cancer*, **7**, 17.

BALDWIN, R. W., EMBLETON, M. J. & MOORE, M. (1973*a*) Immunogenicity of Rat Hepatoma Fractions. *Br. J. Cancer*, **28**, 389.

BALDWIN, R. W., EMBLETON, M. J. & ROBINS, R. A. (1973*b*) Cellular and Humoral Immunity to Rat Hepatoma-specific Antigens Correlated with Tumour Status. *Int. J. Cancer*, **11**, 1.

BALDWIN, R. W. & GLAVES, D. (1972) Solubilization of Tumour-specific Antigen from Plasma Membrane of an Aminoazo-dye-induced Rat Hepatoma. *Clin. & exp. Immunol.*, **11**, 51.

BALDWIN, R. W., GLAVES, D. & PIMM, M. V. (1971*b*) Tumor-associated Antigens as Expressions of Chemically-induced Neoplasia and Their Involvement in Tumor–Host Interactions. In *Progress in Immunology*. Ed. B. Amos. New York: Academic Press. p. 907.

BALDWIN, R. W., GLAVES, D., PIMM, M. V. & VOSE, B. M. (1972*a*) Tumour Specific and Embryonic Antigen Expression on Chemically Induced Rat Tumours. *Ann. Inst. Pasteur*, **122**, 715.

BALDWIN, R. W., GLAVES, D. & VOSE, B. M. (1972*b*) Embryonic Antigen Expression in Chemically Induced Rat Hepatomas and Sarcomas. *Int. J. Cancer*, **10**, 233.

BALDWIN, R. W., GLAVES, D. & VOSE, B. M. (1972*c*) Fetal Antigen Expression on Chemically Induced Rat Neoplasms. In *Embryonic and Fetal Antigens in Cancer*. Ed. N. G. Anderson, J. H. Coggin, E. Cole and J. W. Holleman. U.S.A.E.C., Vol. 2, p. 193.

BALDWIN, R. W., GLAVES, D. & VOSE, B. M. (1974) Immunogenicity of Tumour-associated Embryonic Antigens. *Int. J. Cancer*. In the press.

BALDWIN, R. W., HARRIS, J. R. & PRICE, M. R. (1973*d*) Fractionation of Plasma Membrane-associated Tumour-specific Antigen from an Aminoazo Dye-induced Rat Hepatoma. *Int. J. Cancer*, **11**, 385.

BALDWIN, R. W. & MOORE, M. (1969) Isolation of Membrane-associated Tumour-specific Antigen from an Aminoazo-dye-induced Rat Hepatoma. *Int. J. Cancer*, **4**, 753.

COGGIN, J. H., AMBROSE, K. R. & ANDERSON N. G. (1970) Fetal Antigen Capable of Inducing Transplantation Immunity against SV40 Hamster Tumor Cells. *J. Immun.*, **105**, 524.

COGGIN, J. H., AMBROSE, K. R., BELLOMY, B. B. & ANDERSON, N. G. (1971) Tumor Immunity in Hamsters Immunized with Fetal Tissue. *J. Immun.*, **107**, 526.

COGGIN, J. H. & ANDERSON, N. G. (1972) Phase-specific Autoantigens (Fetal) in Model Tumor Systems. In *Embryonic and Fetal Antigens in Cancer*. Ed. N. G. Anderson, J. H. Coggin, E. Cole and J. W. Holleman. U.S.A.E.C. Vol. 2, p. 91.

HAUGHTON, G. (1966) Transplantation Antigen of Mice: Cellular Localization of Antigen Determined by the H–2 Locus. *Transplantation*, **4**, 238.

LOWRY, O. H., ROSEBROUGH, N. L., FARR, A. L. & RANDELL, R. J. (1951) Protein Measurement with the Folin Phenol Reagent. *J. biol. Chem.*, **195**, 265.

MENARD, S., CALNAGHI, M. I. & DELLA PORTA, G. (1973) *In vitro* Demonstration of Tumor-specific Common Antigens and Embryonal Antigens in Murine Fibrosarcomas Induced by 7, 12-dimethylbenz(*a*)anthracene. *Cancer Res.*, **33**, 478.

THOMSON, D. M. P. & ALEXANDER, P. (1973) A Cross-reacting Embryonic Antigen in the Membrane of Rat Sarcoma Cells which is Immunogenic in the Syngeneic Host. *Br. J. Cancer*, **27**, 35.

TING, C. C., LAVRIN, G. S., SHIV, G. & HERBERMAN, R. B. (1972) Expression of Fetal Antigens in Tumor Cells. *Proc. natn. Acad. Sci. U.S.A.*, **69**, 1664.

TURNER, M. J., STROMINGER, J. L. & SANDERSON, A. R. (1972) Enzymic Removal and Re-expression of Histocompatibility Antigen, H2–A2, at the Surface of Human Peripheral Lymphocytes. *Proc. natn. Acad. Sci. U.S.A.*, **69**, 200.

WITSCHI, E. (1956) *Development of Vertebrates*. London: W. B. Saunders Co.